# Anti-human immunodeficiency virus-1 activity of MoMo30 protein isolated from the traditional African medicinal plant *Momordica balsamina*

**DOI:** 10.1186/s12985-023-02010-5

**Published:** 2023-03-22

**Authors:** Mahfuz Khan, Amad Diop, Erick Gbodossou, Peng Xiao, Morgan Coleman, Kenya De Barros, Hao Duong, Vincent C. Bond, Virginia Floyd, Kofi Kondwani, Valerie Montgomery Rice, Sandra Harris-Hooker, Francois Villinger, Michael D. Powell

**Affiliations:** 1 Department of Microbiology, Biochemistry, and Immunology, Morehouse School of Medicine, 720 Westview Dr. SW, Atlanta, GA 30310 USA; 2 Malango Traditional Healers Association, Fatick, Senegal; 3 PROMETRA International, BP 6134, Dakar-Etoile, Senegal; 4 Department of Pharmacology, Morehouse School of Medicine, 720 Westview Dr. SW, Atlanta, GA 30310 USA; 5 Department of Community Health and Preventive Medicine, Morehouse School of Medicine, 720 Westview Dr. SW, Atlanta, GA 30310 USA; 6 Office of the President, Morehouse School of Medicine, 720 Westview Dr. SW, Atlanta, GA 30310 USA; 7 Department of Pathology Senior Vice President for External Affairs and Innovation, Morehouse School of Medicine, 720 Westview Dr. SW, Atlanta, GA 30310 USA; 8 Department of Biology Director, New Iberia Research Center, University of Louisiana at Lafayette, 4401 W Admiral Doyle Drive, New Iberia, LA 70560 USA

**Keywords:** MoMo30-plant, Anti-viral, gp120, HIV-1, Glycan, CBA, Momordica, Lectin

## Abstract

**Background:**

Plants are used in traditional healing practices of many cultures worldwide. *Momordica balsamina* is a plant commonly used by traditional African healers as a part of a treatment for HIV/AIDS. It is typically given as a tea to patients with HIV/AIDS. Water-soluble extracts of this plant were found to contain anti-HIV activity.

**Methods:**

We employed cell-based infectivity assays, surface plasmon resonance, and a molecular-cell model of the gp120-CD4 interaction to study the mechanism of action of the MoMo30-plant protein. Using Edman degradation results of the 15 N-terminal amino acids, we determined the gene sequence of the MoMo30-plant protein from an RNAseq library from total RNA extracted from *Momordica balsamina*.

**Results:**

Here, we identify the active ingredient of water extracts of the leaves of *Momordica balsamina* as a 30 kDa protein we call MoMo30-plant. We have identified the gene for MoMo30 and found it is homologous to a group of plant lectins known as Hevamine A-like proteins. MoMo30-plant is distinct from other proteins previously reported agents from the Momordica species, such as ribosome-inactivating proteins such as MAP30 and Balsamin. MoMo30-plant binds to gp120 through its glycan groups and functions as a lectin or carbohydrate-binding agent (CBA). It inhibits HIV-1 at nanomolar levels and has minimal cellular toxicity at inhibitory levels.

**Conclusions:**

CBAs like MoMo30 can bind to glycans on the surface of the enveloped glycoprotein of HIV (gp120) and block entry. Exposure to CBAs has two effects on the virus. First, it blocks infection of susceptible cells. Secondly, MoMo30 drives the selection of viruses with altered glycosylation patterns, potentially altering their immunogenicity. Such an agent could represent a change in the treatment strategy for HIV/AIDS that allows a rapid reduction in viral loads while selecting for an underglycosylated virus, potentially facilitating the host immune response.

**Supplementary Information:**

The online version contains supplementary material available at 10.1186/s12985-023-02010-5.

## Introduction

Plants are the source of treatments for a wide variety of diseases worldwide [1–4]. This includes viral infections such as human immunodeficiency virus type 1 (HIV-1) [3, 5]. Many medicinal plants can be found in the family *Cucurbitaceae* [6, 7], including the genus *Momordica*. The best-known medicinal plant in *Momordica* is the species *Momordica charantia*, commonly called "bitter melon." Bitter melon is used to treat a variety of medical conditions, from chronic inflammation [8] and diabetes [9–11] to cancer [12, 13]. It has also been reported to have various inhibitory effects on viruses, bacteria, and parasites (see [14]). *Momordica* anti-HIV protein (MAP30), a ribosome-inactivating protein (RIP) isolated primarily from the seeds of *Momordica charantia,* has been shown to have anti-HIV activity [15, 16]. RIPs are N-glycosidases that depurinate ribosomal ribonucleic acid (rRNA) [17]. This depurination irreversibly inactivates the ribosome, blocking protein synthesis [18]. Another species within the *Cucurbitaceae* is *Momordica balsamina.* It also has been reported to produce a RIP with anti-HIV-1 activity [19, 20]. The anti-HIV-1 activity of RIPs has been attributed to their interaction with viral nucleic acid and interaction with a post-reverse transcription step in replication [19]. *Momordica balsamina* is also used to treat other medical conditions, such as gastric ulcers [21, 22].

Proteins that bind to carbohydrate residues are collectively called carbohydrate-binding agents (CBAs) [23]. CBAs can block the binding of envelope glycoproteins with their target receptors on cells. The ability of CBAs to bind to glycoproteins and block their interaction with receptors has been proposed as a potential means to inhibit enveloped viruses like HIV-1 [23–26]; however, to date, no agent has become commercially available. A common type of CBA found in plants is lectins, which can bind to glycoproteins and cause red blood cells to agglutinate [26, 27]. Plant Lectins are reportedly more resistant to heat denaturation than animal proteins [28]. Indeed, banana and other plant lectins have been proposed to have various anti-microbial functions, including the ability to inhibit HIV-1 and Influenza viruses [27]. It has been theorized that treatment of HIV-1 with CBAs could select for mutations that leave "holes" in the carbohydrate layer and allow for broadly neutralizing antibodies to be induced [29]. Therefore, there has been significant interest in finding plant CBAs that could potentially be used to treat HIV/AIDS. Chitinases are another ubiquitous group of plant proteins that catalyze the breakdown of chitin found in the cell walls of fungi and can provide protection against fungal infection [30]. Some plant chitinases have evolved the ability to hemagglutinate red blood cells and are sometimes called chi-lectins [31]. Therefore, some of the many chitinases from plants could function as CBAs and potentially be used as antiviral treatments. Hevamine A-like proteins are a type of chitinase that generally act as plant defensins protecting the plant from pathogens with chitin in their cell walls, such as fungi [32].

This report details the characterization of a 30 kDa protein we have previously reported [33] from the medicinal plant *Momordica balsamina* used by traditional healers in certain African regions. Its N-terminal sequence has homology to Hevamine A-like proteins in other plants. It can bind to HIV-1 gp120 and inhibit the virus. It is a CBA and could represent a potential new type of therapy against HIV that would allow a short-term treatment to induce long-term viral suppression.

## Results

### The active agent of plant extracts is a 30 kDa protein

**Fig. 1:**
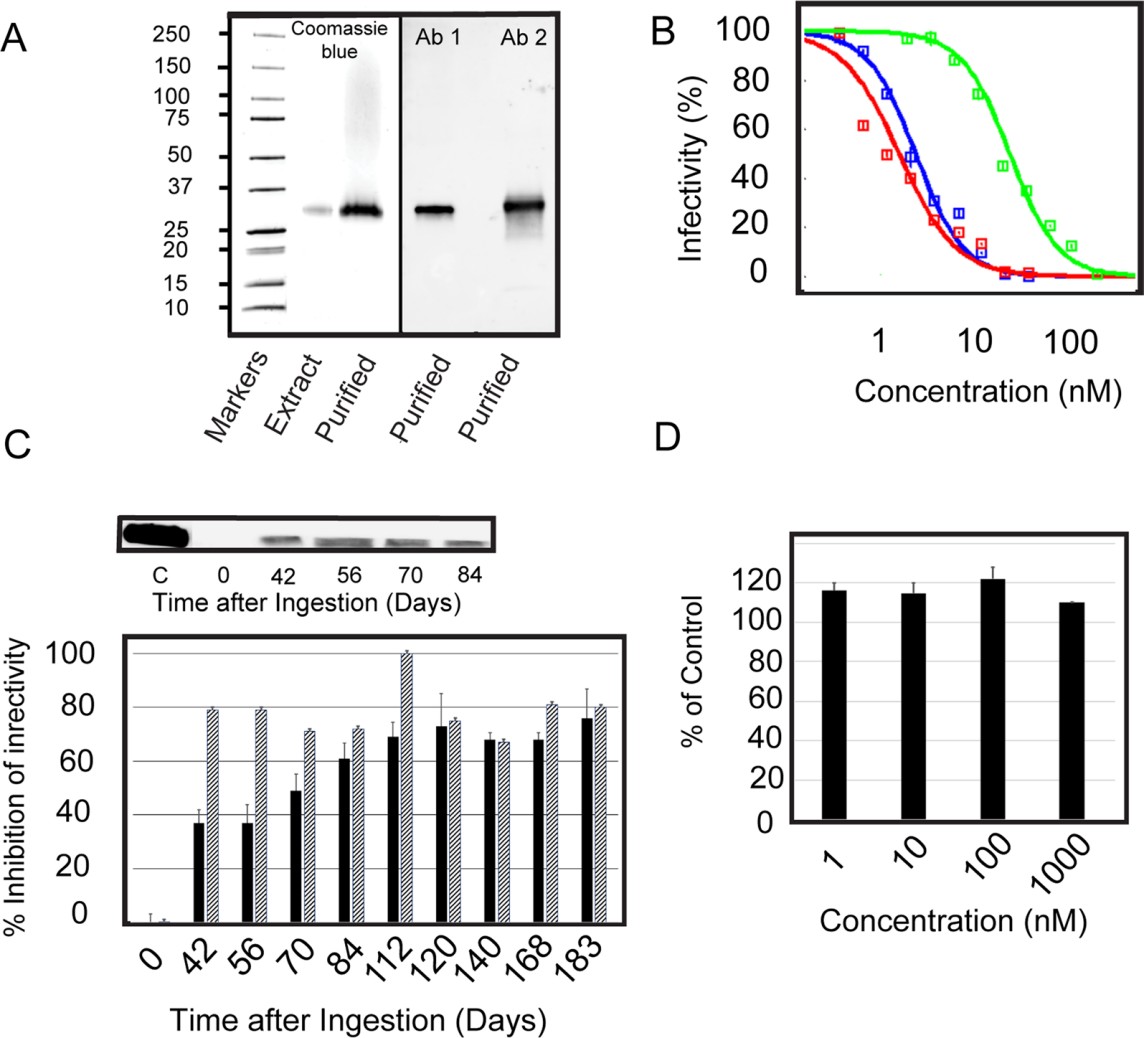
Plant extracts primarily contain a 30 kDa protein as visualized by Coomassie stain on a 4–20% SDS-PAGE gel. **A** A Coomassie blue stained gel of the extract and extract passed through a 30kD cutoff filter shows a predominantly single band. The band is reactive with an N-terminal antibody to the MoMo30-plant (Ab 1) and a second antibody made from a sequence from the gene of the predicted MoMo30-plant (Ab 2) **B**. The IC_50_ of the protein was determined by exposing HIV-1_NL43_ (equivalent to 1 ng p24) to concentrations of MoMo30-plant from 1 to 100 nM and determining the percent infectivity by MAGI assay. The IC_50_ value was determined by curve-fitting using Dr. Fit software. The blue curve is MoMo30-plant, and the red curve is MoMo30-HEK. The green curve is the commercially available fusion inhibitor Enfuvirtide. **C** MoMo30-plant is adsorbed into the serum of Rhesus macaques. Two macaques were given herbal therapy in the same regimen as in the field for humans (adjusted for weight). Three microliters of serum were tested by MAGI assay (in triplicate) for antiviral effects from 0 to 183 days. The inset shows a western blot using N-terminal ab and 15 µL of the sample in crosshatched bars. **D** MoMo30-plant shows no cellular toxicity at therapeutic levels. MoMo30-plant was tested at concentrations from 1 to 1000 nm in an MTT assay. Control is untreated cells

We have previously shown that extracts of *Momordica balsamina* made in water contain antiviral activity [33]. The active agent is a 30 kDa protein we call MoMo30. Hereafter called MoMo30-plant to denote plant-derived protein. A representative extract is shown on a 4–20% SDS-PAGE gel. It shows a single band of approximately 30 kDa in size (see Fig. 1A) in the original extract and after concentration on a 30 kDa cutoff filter (Fig. 1A, Purified). Surprisingly, wedetected no other significant bands on the SDS-PAGE gel. We used molecular weight cutoff filters to separate MoMo30-plant from lower molecular weight contaminants and to concentrate the protein. The N-terminal sequence of the first 15 amino acids was determined by Edman degradation and was found to be: GPIVTYWGQNVXEGEL. Western blot analysis with an antibody directed against the N-terminal peptide confirmed that the protein subjected to Edman degradation is indeed the 30-kDa band detected by Coomassie Blue staining of polyacrylamide gels (Fig. 1A, Ab 1). MoMo30-plant was also reactive with a second peptide antibody made from an internal region of the predicted gene sequence for Hevamine A-like protein (Fig. 1A, Ab 2).

### MoMo30-plant can inhibit both X4 and R5 tropic strains of HIV-1

We tested the purified MoMo30-plant protein for its ability to inhibit HIV-1 infection in a MAGI cell assay (see Fig. 1B). Purified protein was able to inhibit HIV-1_NL4-3_ in a dose-dependent fashion. We tested concentrations of MoMo30-plant from 1 to 1000 nM. The IC_50_ of the protein was determined by curve fitting using the program Dr. Fit as described [34] and was determined to be 2.8 nM (Fig. 1B). The plot was curve-fit from triplicate measurements of two independently isolated purified protein preparations (blue curve). For comparison, we also ran a dose-dependence curve on MoMo30-HEK (red curve). Triplicate sample analysis of the commercially available fusion inhibitor Enfuvirtide was also conducted in parallel (Fig. 1B green curve). The IC_50_ of Enfuvirtide was determined to be 44 nM, consistent with the previously published value of 26 nM [35].

To determine the ability of MoMo30-plant to inhibit strains of HIV-1 other than HIV-1_NL4-3,_ we tested a panel of primary isolates from different clades and origins. The isolates included HIV-1_IIIb_, HIV-1_JRFL_, HIV-1_93/MW/965_, HIV-1_97/ZA/012_, HIV-1_94/UG/118_, HIV-1_92/RW/024_, and HIV-1_93/RW/002_ and the results are summarized in Table 1 and the representative IC_50_ values are shown. The IC_50_ values ranged from 2.8 to 9.0 nM.

**Table 1:** Inhibition of primary isolates

Strain	Clade	IC50 (nM)
HIV-1 IIIb	B	0.7 ± 0.03
HIV-1 92/RW/024	A	4.6 ± 0.20
HIV-1 93/RW/002	A	2.0 ± 0.06
HIV-1 JRFL	B	1.7 ± 0.001
HIV-1 93/MW/965	C	3.9 ± 0.001
HIV-1 97/ZA/012	C	9.0 ± 0.02
HIV-1 94/UG/118	D	8.1 ± 0.07
HIV-1 NL43	B	2.8 ± 0.001

### Orally delivered MoMo30-plant can accumulate in the bloodstream of non-human primates

To determine if orally administered plant extract resulted in the presence of MoMo30-plant in the bloodstream, we administered extracts of the medicinal plants to two *Rhesus macaques* by mouth for six months. We did MAGI infectivity assays and Western blot to confirm the presence of MoMo30-plant in the serum of treated animals (Fig. 1C). Serum was tested from 0 to 183 days after ingestion. Analysis of samples of one of the animals by Western blot using the antibody specific for the MoMo30-plant N-terminus showed that MoMo30-plant remains in the bloodstream for at least 84 days Fig. 1C, inset). Neither animal had detectable protein present in their serum before ingestion. Neither animal exhibited inhibitory activity in their serum prior to ingesting the plant extract; however, sera from both possessed significant amounts of antiviral activity by 42 days post-ingestion (Fig. 1C).

### MoMo30-plant *is not toxic to cells over its inhibitory range*

We tested the cellular toxicity of the MoMo30-plant using the MTT assay. We tested concentrations of MoMo30-plant from 1 to 1000 nM. Over this range, the protein showed minimal cellular toxicity (see Fig. 1D).

### MoMo30-plant stability studies

**Fig. 2:**
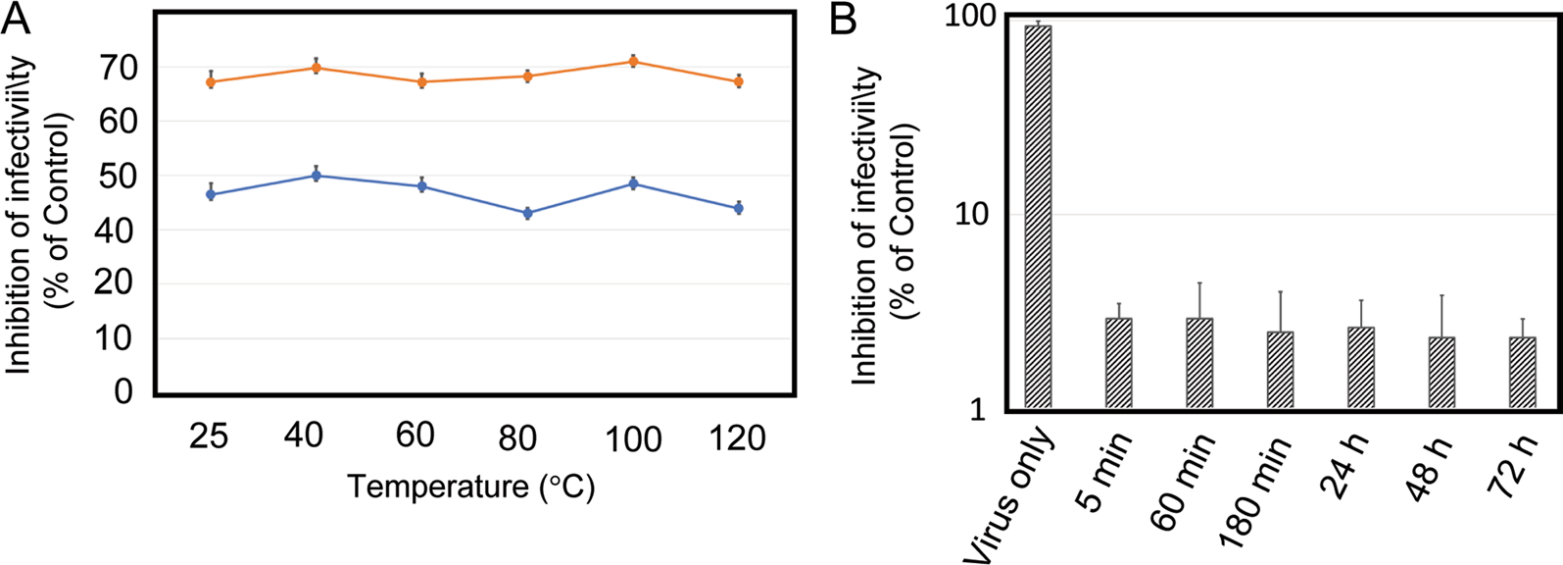
MoMo30-plant is heat stable and stays bound to the virus for long periods. **A** MoMo30-plant was mixed with HIV-1_NL43_ (equivalent to 1 ng p24) at concentrations of 0.1 (red) and 1 (blue) nM sufficient to cause 50% (blue) or 70% inhibition (red). The mixture was then heated at temperatures from 25° to 120° for 30 min, allowed to cool to 25 °C. An aliquot of 100 µL was then tested in the MAGI infectivity assay, **B** MoMo30-plant (3 nM) was mixed with HIV-1_NL43_ (equivalent to 1 ng p24) and allowed to interact for 5 min before centrifuging the complex through a 40% sucrose cushion to remove from free MoMo30-plant. The virus-MoMo30-plant complex (in the pellet) was removed from 5 min to 72 h at 4 °C before testing by MAGI cell assay. All measurements were done in triplicate

We studied the heat stability of MoMo30-plant by testing the ability to inhibit HIV infectivity after incubation of the protein at temperatures from 25 to 120 °C (autoclaving). The activity of the MoMo30-plant was tested at concentrations of 1 nM (red line), sufficient to inhibit the virus by 70%, and 0.1 nM (blue line), sufficient to inhibit the virus by approximately 50%. The results are summarized in Fig. 2A. We found that over a range from 25 to 120 °C, the percent infectivity of the purified protein remained relatively unchanged (Fig. 2A). To determine the stability of MoMo30-plant complexes formed with HIV-1, we mixed 100 ng/mL of MoMo30-plant with HIV-1_NL4-3_ virus equivalent to 1 ng of p24. Typically 1 ng of p24 will produce 10,000 ± 150 blue cells/ng p24 using HIV-1_NL4-3,_ and we include a virus-only control in each experiment. The virus plus MoMo30-plant was centrifuged through a 30% sucrose cushion at 125,000×*g* to remove any unbound MoMo30-plant. The sucrose pellet containing virus plus bound MoMo30-plant was then tested for infectivity by MAGI cell assay at 5 min to 3 days intervals. The results are summarized in Fig. 2B. Complexes of MoMo30-plant and the virus retained 100% of their antiviral activity for at least 72 h, suggesting that the complex of MoMo30-plant and virus remains stable once formed.

### Comparison of the N-terminal sequence of MoMo30-plant to other proteins

**Fig. 3:**
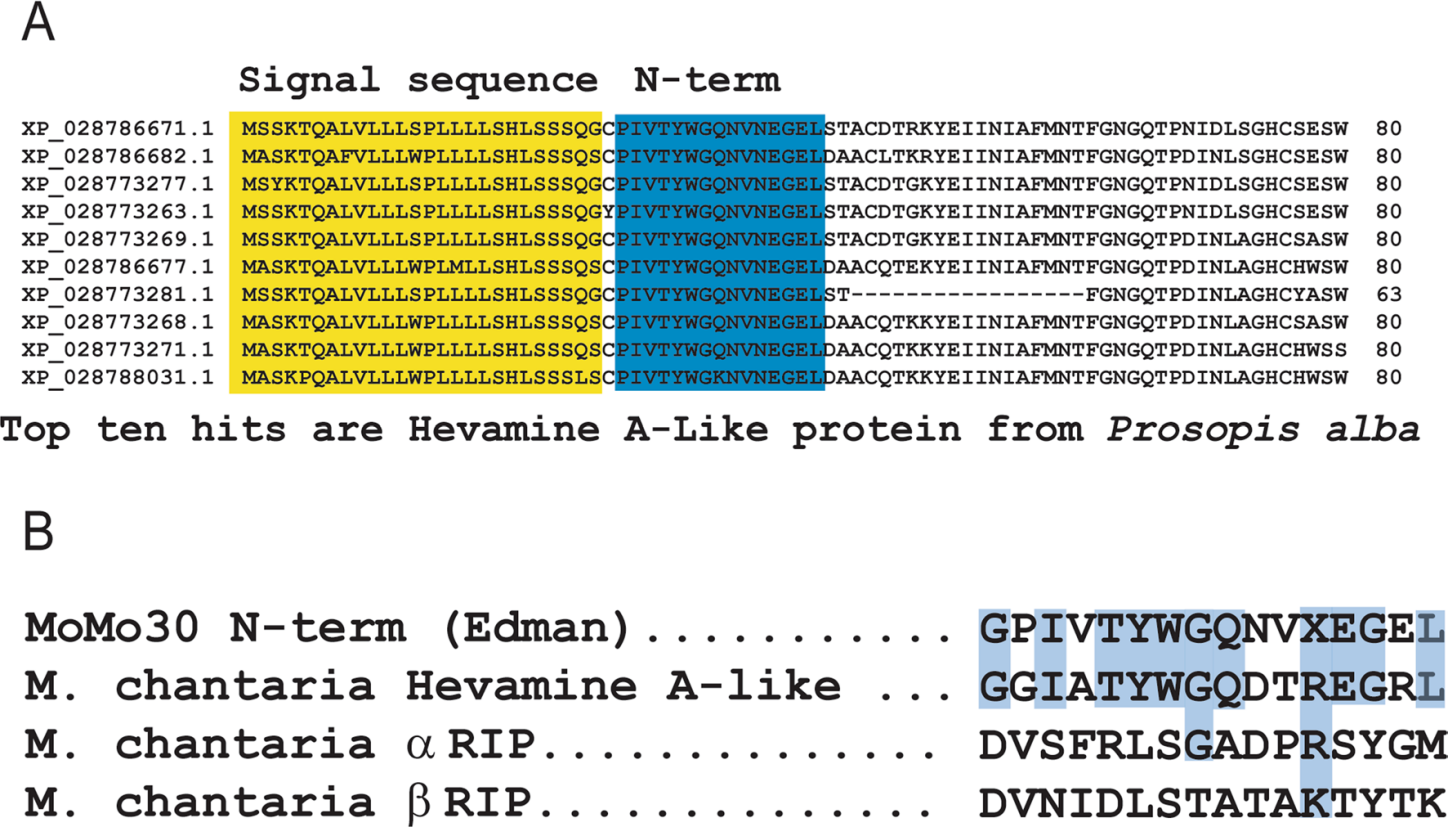
MoMo30-plant is homologous to the Hevamine A-like protein. **A** The N-terminal sequence from MoMo30-plant was used to search protein BLAST. The top ten hits are shown. The yellow box highlights the signal sequence, and the blue highlights the homologous portion of the N-terminal sequence. **B** The N-terminal sequence from MoMo30-plant was compared to the Hevamine A-like protein from M. chantaria and the ribosome-inactivating protein (RIP) from *M. chantaria* both alpha and beta forms. Conserved amino acids are highlighted in blue

To help determine the identity of the MoMo30-plant protein, the sequence of the N-terminal 15 amino acids was determined by Edman degradation (GPIVTYWGQNVXEGEL). The Edman sequence was compared to other proteins in the NR (NCBI) database using the BLAST algorithm (Fig. 3A). The top ten hits on the database were all forms of the Hevamine A-like protein from *Prosopis alba*, the South American (white carob tree) plant defensin with chitinase activity similar to those described above [32]. We compared the N-terminal sequence to Momordica's previously reported RIP protein [15, 36] (Fig. 3B). The N-terminal sequence from MoMo30-plant had no homology to the RIP proteins from *M. charantia *both the α and β momorcharins. In contrast, the N-terminal sequence from MoMo30-plant had the N-terminal sequence from MoMo30-plant had significant homology with the Hevamine A-like protein from M. charantia (Fig. 3B). The high degree of homology suggests that MoMo30-plant could be an unreported Hevamine A-like protein from *M. balsamina*.

### RNAseq analysis of *Momordica balsamina* reveals significant homology to the Hevamine A-like gene

**Fig. 4:**
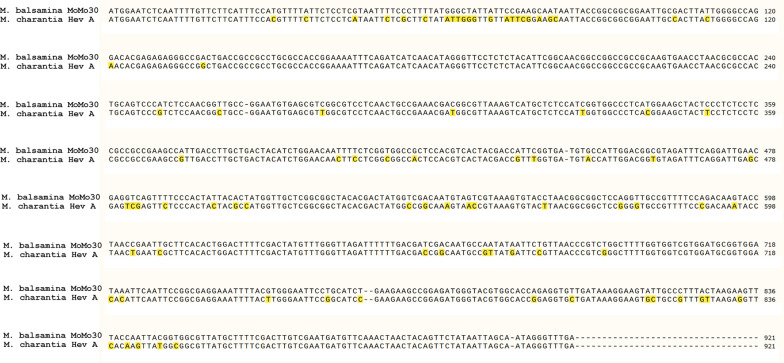
Clustal omega alignment of DNA sequences from MoMo30-plant, Hevamine A-like protein from *M. charantia*. Nucleotides that differ from the MoMo30-plant sequence are highlighted in yellow. MoMo30-plant is 92% identical to the M. charantia Hevamine A-like gene

We obtained enough RNA from freshly grown *M. balsamina* plant cells to obtain RNAseq data from the de novo transcriptome. A Diamond BLAST search of open reading frames identified in the sequence revealed homology between the N-terminal sequence of the MoMo30-plant protein and a Hevamine A-like sequence translated from the RNAseq data though it was not identical. The complete gene sequence assembled from RNAseq reads is shown in Fig. 4. The gene was 93% identical to the Hevamine A-like gene from *M. chantaria*. Therefore, we considered that MoMo30-plant is a previously unreported Hevamine A-like protein from *M. balsamina*. A second peptide antibody was made in rabbit using a peptide sequence derived from the Hevamine A-like gene sequence and was reactive with the MoMo30-plant protein (Fig. 1A; Ab2).

**Fig. 5:**
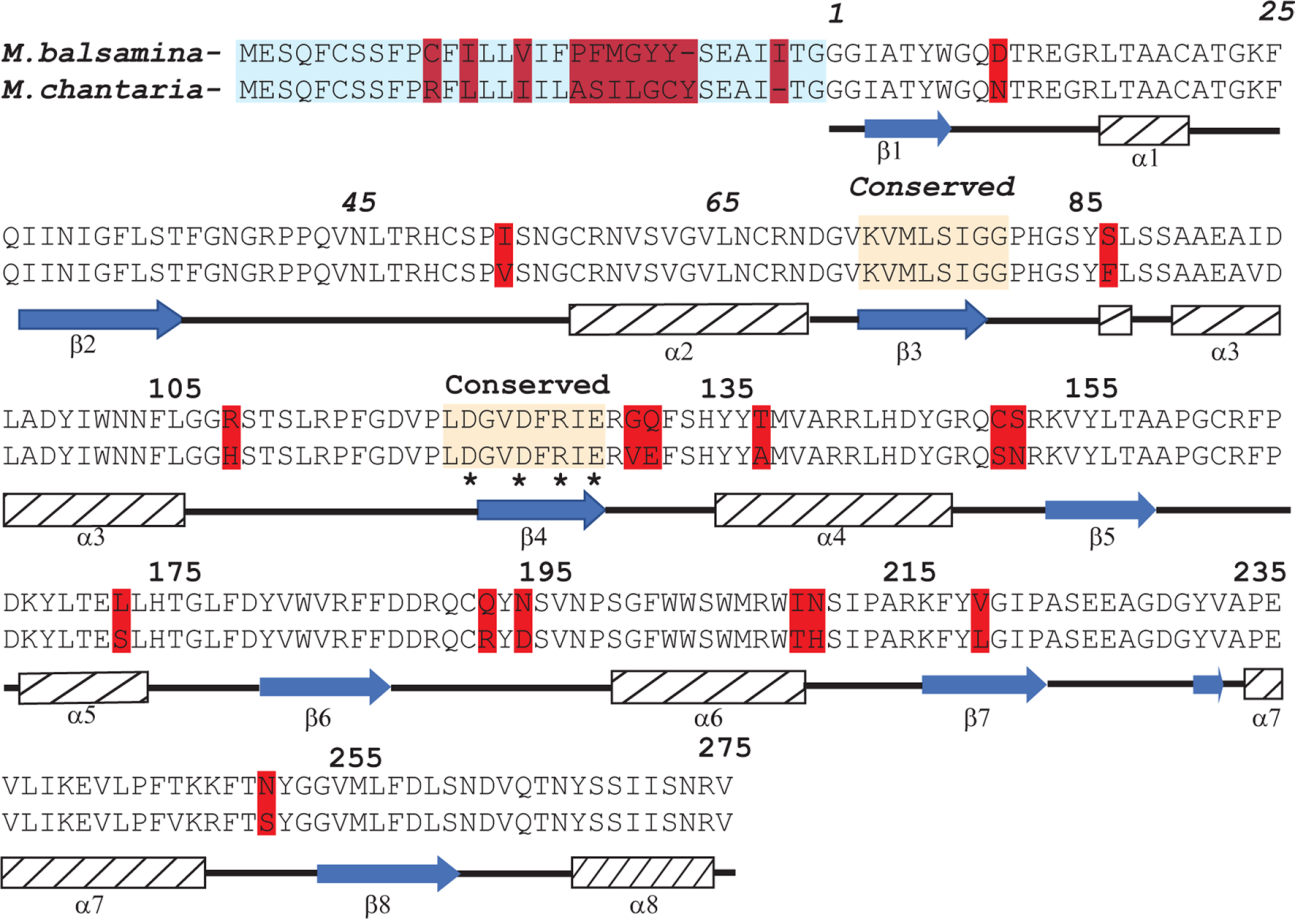
Translation of the DNA sequence of MoMo30-plant, including structural prediction of the resulting protein. Amino acids highlighted in red are differences between the two sequences. Arrows denote the predicted beta-sheet structure, and hatched boxes denote predicted alpha-helical structure. The two yellow shaded boxes denote domains of conservation in this class of proteins. The asterisks denote the highly conserved catalytic residues

A translation of this gene sequence and secondary structure prediction (by the Phyre2 website) is shown in Fig. 5. The predicted secondary structure shows strong homology to a TIM β-barrel (a structure that is commonly found in Hevamine A-like proteins [37]. The TIM structure is reported to be a very heat-stable conformation [38], consistent with our observation that MoMo30-plant is heat-stable (see Fig. 3).

### In vitro transcription/translation of the Hevamine A-like MoMo30-plant gene produces an antiviral effect

**Fig. 6:**
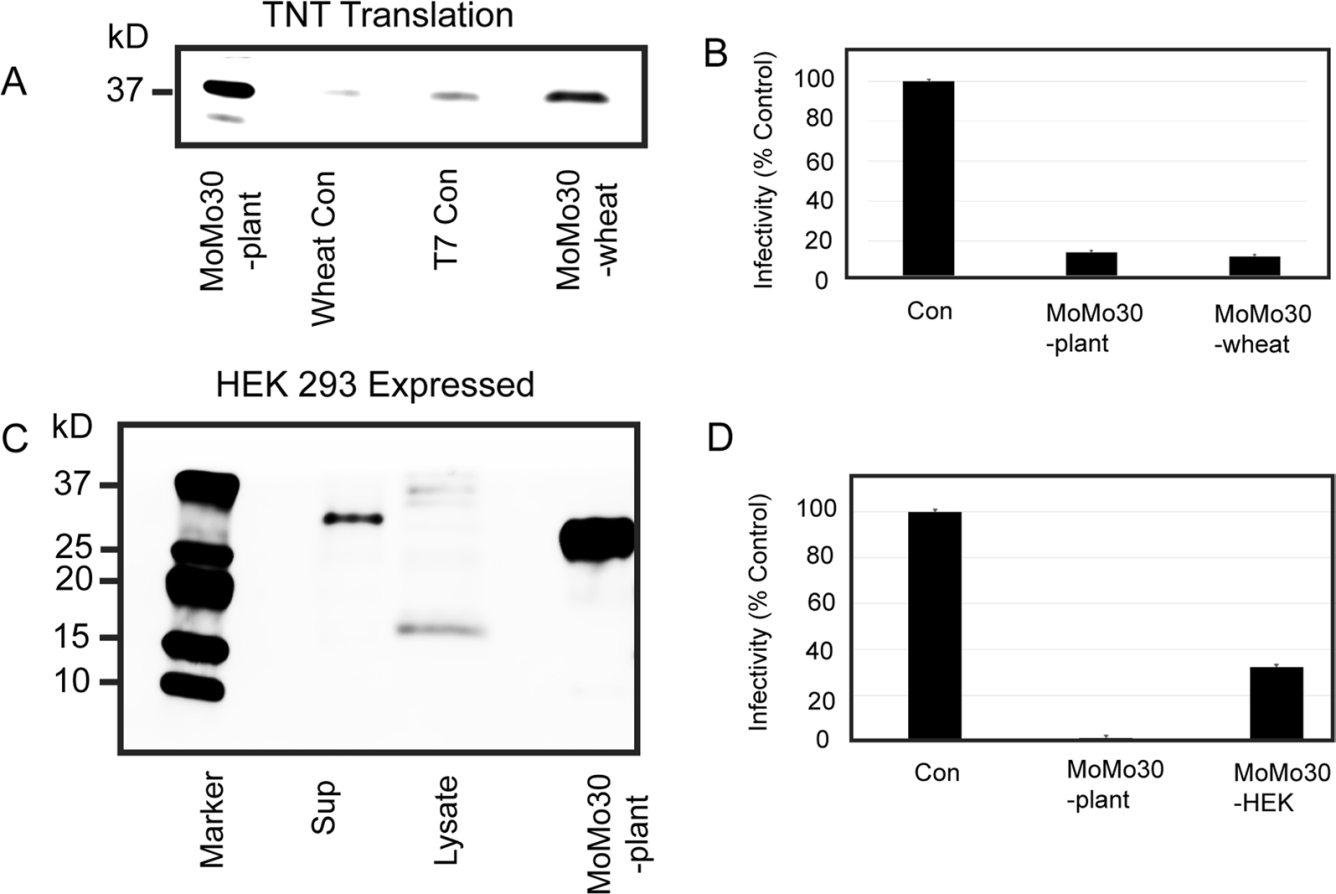
In vitro translation of the MoMo30-plant gene produces a 30 kD protein with antiviral activity. **A** An in vitro synthesized gene was inserted into a pGenLenti vector and used as a template for coupled transcription/translation. The reaction was run on a 20% SDS-PAGE gel, and a western blot was probed with an N-terminal ab to MoMo30-plant. **B** One hundred µL of the reaction mix was tested in a MAGI assay. **C** The MoMo30-plant pGenLenti plasmid was used to transfect HEK 293 cells. Supernatant and cell lysates were run on a 20% SDS-PAGE gel and probed with the N-terminal ab. A sample of purified MoMo30-plant is used as a marker. **D** One hundred µL of the cell-free conditioned medium as tested by MAGI assay

We took the sequence of the MoMo30-plant gene derived from the RNAseq data and had the gene synthesized and cloned into the pGen-lenti vector expression plasmid. In vitro coupled transcription and translation were done on purified template using the TNT wheat germ extract system (Fig. 6A). The product of the reaction was tested by MAGI assay to determine if there was any antiviral effect. The synthesized product MoMo30-wheat (panel A) inhibited HIV-1 similarly to purified MoMo30-plant (see Fig. 6B). Western blot revealed an ~ 30 kDa protein in the reaction. We transfected HEK-293 cells with the MoMo30-plant plasmid and tested the cell-free supernatant and cell lysate by western blot using an N-terminal antibody (Fig. 6B). We detected a single 30 kDa band in the supernatant. It cannot be explained why no processed MoMo30 is present in the cell lysate. We tested the cell-free culture supernatant for antiviral activity by the MAGI assay (panel D) and found that tissue culture supernatants could inhibit HIV-1 infection significantly, and a dose–response curve was similar to that seen for the MoMo30-plant protein (Fib 1B, red curve). In addition, Hevamine A-like proteins are known to have chitinase activity. MoMo30-HEK had chitinase activity (see Additional file 1: Fig S1). Finally, MoMo30-HEK could retain its anti-HIV activity after 30 min of incubation at 100 °C (Fig. 1B, the red curve is tested on heated protein). Together, these data suggested that the MoMo30-HEK protein was a Hevamine A-like protein from *M. balsamina*.

### MoMo30-plant can block the binding of gp120 to Jurkat cells

**Fig. 7:**
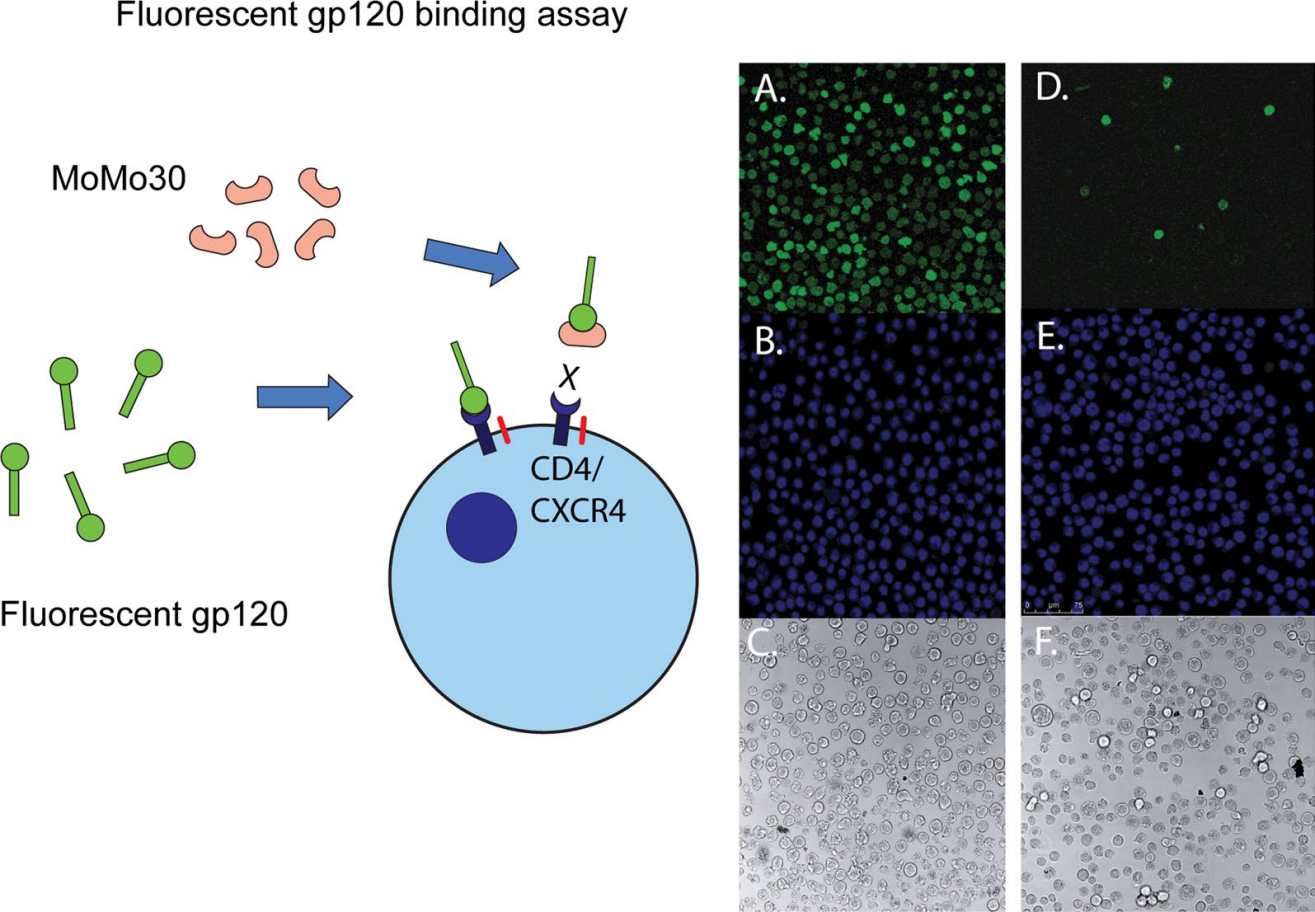
MoMo30-plant can bind to purified FITC labeled gp120 and blocks its interaction with Jurkat cells. **A** Labeled gp120 was added to Jurkat cells and allowed to bind to the surface, making it visible. **B** The same cells were stained with Hoechst 33342 nuclear stain. **C** Phase contrast image. **D** Pre-incubation of fluorescent gp120 was done with a stock of 200 µg of MoMo30-plant, which blocks its interaction with the cell. **E** The same cells were stained with Hoechst 33342 nuclear stain. **F** Phase contrast image

**Fig. 8:**
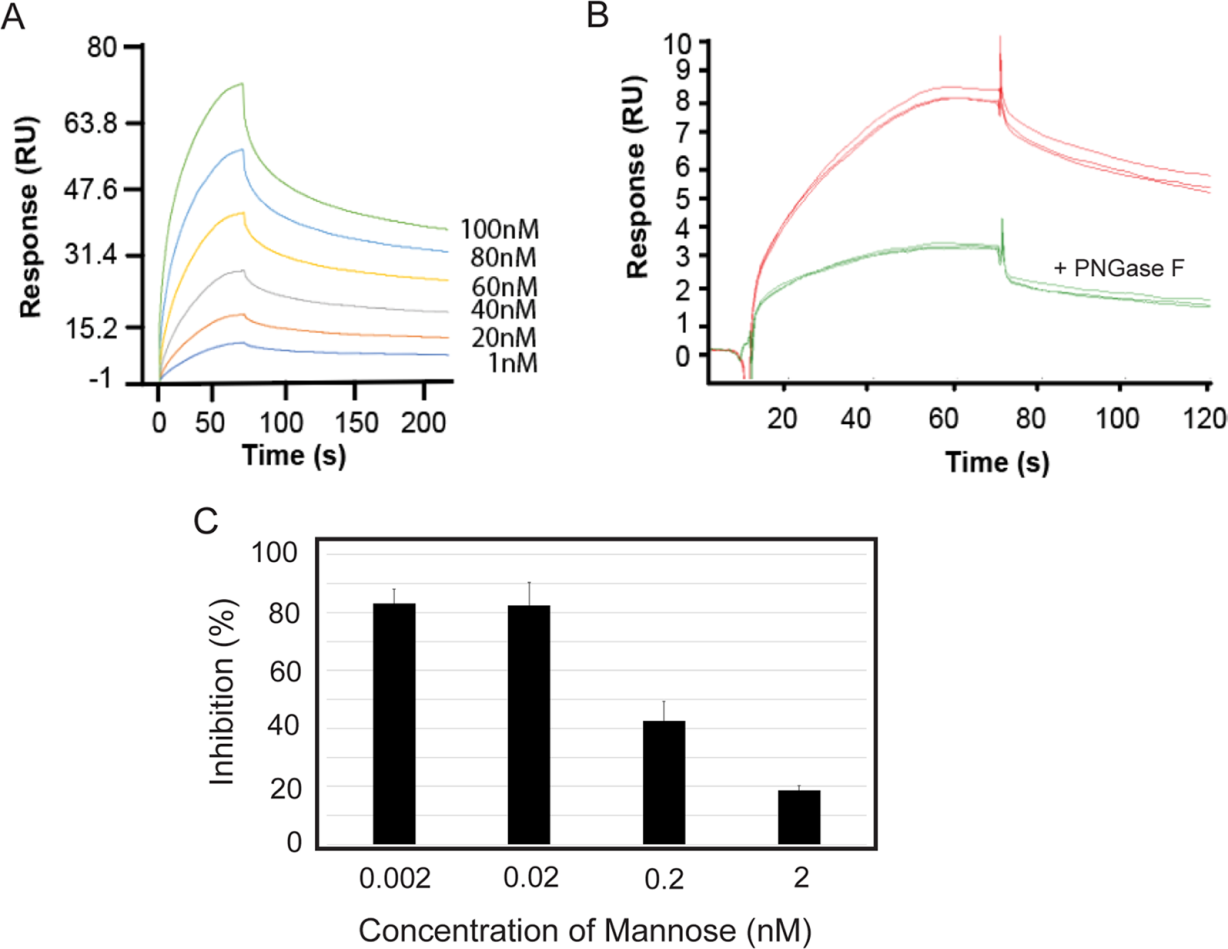
MoMo30-plant binds to purified gp120. **A** Gp120 was bound to a Biacore chip surface, and MoMo30-plant was allowed to flow across the chip at concentrations from 1 to 100 nM. Changes in surface plasmon resonance monitored binding. **B** Gp120 pre-treated with PNGase F (an N-linked glycosylase) dramatically reduces binding. The three lines represent triplicate measurements. **C** Mannose can block the activity of MoMo30-plant. HIV-1_NL4-3_, 2 nM of MoMo30-plant, and different concentrations of D-mannose were added simultaneously and incubated for 5 min at 37 °C before testing by MAGI cell assay for inhibition of infection. Inhibition as a percentage of the untreated control (Infection in the presence of 2 nM Momo30-plant) is plotted

To determine the stage of viral replication inhibited by the MoMo30-plant, we did assays that modeled steps in replication. To assay the attachment of gp120 to susceptible cells, we used purified FITC labeled gp120 mixed with Jurkat cells. FITC-labeled gp120 ornaments the cell surface making it visible. In the absence of MoMo30-plant, the purified gp120 can attach to CD4 or CXCR4 on the surface of Jurkat cells (see Fig. 8A–C). A stock solution of 200 nM of MoMo30-plant (sufficient to completely inhibit virus equivalent to 1 ng of p24) was pre-incubated with the gp120 before adding the Jurkat suspension. Treatment with MoMo30-plant blocked the interaction of gp120 with Jurkat cells (compare Fig. 7A–D). This finding suggests that MoMo30-plant blocked the initial step in replication by binding to gp120 and blocking entry.

### MoMo30-plant-plant binds to the glycan residues of gp120

We did surface plasmon resonance experiments to quantify the interaction of MoMo30-plant-plant with gp120 better. Purified gp120 was bound to a chip surface, and MoMo30-plant-plant was allowed to flow across the surface, allowing binding to be characterized (Fig. 8A). Concentrations of MoMo30-plant-plant from 1 to 100 nM were analyzed. Increasing concentration of MoMo30-plant-plant showed proportional increases in changes in reflectance of the chip (Fig. 8A). Three independent measurements in triplicate gave a kd of 2.05 × 10^–3^ 1/s, KD of 3.0 nM, and ka of 6.923 × 10^5^ 1/Ms. The binding profile suggests that there is a fast on rate and a biphasic off rate. The initial dissociation is rapid, followed by a very slow dissociation. We speculate that the fast part of this off rate may be due to the dissociation of multimers (see Additional file 1: Fig. S2) followed by a slow dissociation of the MoMo30-plant complex. To further characterize the binding of MoMo30-plant-plant to gp120, we pre-treated purified gp120 with PNGase F, which removes N-linked glycans. After treatment with PNGase F, we saw a dramatic decrease in binding to the chip surface (Fig. 8B), suggesting that MoMo30-plant binds to gp120 through its glycan residues.

### Effect of monosaccharide mannose on inhibition of infection by MoMo30-plant-plant

The glycans on gp120 are a mixture of high mannose and complex glycans [39, 40]. To determine the effect of adding the monosaccharide mannose to the MoMo30-plant, we investigated inhibition by MoMo30-plant in the presence of different concentrations of mannose (Fig. 8C). We tested inhibition of HIV-1_NL4-3_ by MoMo30-plant (2 nM) in the presence of concentrations of mannose from 0 to 2 nM. We saw that the inhibition of HIV_NL4-3_ by MoMo30-plant was significantly reduced by mannose in a concentration of 2 nM, a 1:1 molar ratio with MoMo30-plant.

## Discussion

In this study, we report the anti-HIV-1 activity of water-soluble plant extracts from the medicinal plant *Momordica balsamina*. We find that the biological activity is contained in a 30 kDa protein we call MoMo30-plant. Anti-HIV-1 activity has been previously reported in extracts of *Momordica balsamina* [20]. A 30 kDa ribosome-inactivating protein (RIP) has been reported that acts at a post-reverse transcription step in replication. MoMo30-plant is distinguishable from RIPs in several ways; first, its mechanism of action appears to be at the stage of attachment and fusion, as evidenced by MoMo30-plant's ability to bind to gp120 and block its interaction with cells expressing CD4 receptors. Second, from studies we did using surface plasmon resonance, MoMo30-plant appears to react specifically with glycans on the surface of gp120 (see Fig. 8). We also find that MoMo30-plant has chitinase activity (see Additional file 1: Fig. S1). Finally, its N-terminal sequence does not share homology with the N-terminal region of known RIPs from Momordica (Fig. 3B). In the current study, MoMo30-plant was isolated from water-soluble extracts made from dried leaves. In contrast, RIPs were isolated primarily from the plant's seeds. The healers partially process the dried leaves of *M. balsamina* by making tea in boiling water. The exceptional heat stability of MoMo30-plant-plant may allow it to survive this process, while any other proteins in the solution would tend to denature and precipitate out of the solution. Heat stability could account for our observation that water-soluble extracts of *M. balsamina* contain a single protein of 30 kDa. Therefore, we consider that MoMo30-plant protein is a previously undescribed protein present in *Momordica balsamina* but distinct from RIP.

The MoMo30-plant protein has been difficult to study using typical proteomics techniques, and the genomic sequence of *M. balsamina* has not been reported. Attempts to do de novo sequencing of the protein were not successful. The exceptional stability of MoMo30-plant may contribute to its relative difficulty digesting with proteolytic digest by traditional enzymatic methods. Repeated attempts to analyze the protein sequence by LC/MS yielded very few or no peptides for analysis. Even intact mass measurements by MALDI/TOF were impossible as MoMo30-plant does not appear to be ionized using various matrix types. However, we obtained 15 amino acids from the N-terminus by Edman degradation (GPIVTYWGQNVXEGEL). Because of our difficulty with proteomics methods, our strategy was to isolate total cellular RNA from fresh plant tissue, perform RNAseq, and then search the transcriptome for proteins that match the N-terminal sequence.

We could germinate seeds and obtain enough growth to isolate total plant RNA. We used this to perform RNAseq (Illumina) and obtain a de novo assembly of the total transcriptome of *M. balsamina*. A Diamond BLAST search for translated proteins showed significant homology to the Hevamine A-like protein from *M. charantia* (see Fig. 4B). There are several possibilities why the match was not exact. The plants used for protein isolation were from Senegal and were dried. To have fresh plant material for RNA isolation, we needed to germinate seeds in our lab under artificial conditions to obtain enough plant cells for processing. Since the two sources of plant material were from different sources and conditions, this may have introduced variation from the mRNA to the original protein sequence. Another possibility is that the sequence identified by Edman determination is different than the gene identified by RNaseq. Both tissue-specific and temporal differences have been reported in the expression of defensin-like genes in Arabidopsis and Medicago [41]. The complete genome of *Momordica balsamina* has not been published to date so we can not rule out that such tissue-specific variants of the Hevamine A-like are present in *Momordica balsamina*. To help ensure we had isolated the correct gene sequence, we had the gene for MoMo30-plant synthesized (GenScript) and cloned into an expression vector. In vitro*,* transcription/translation by TNT wheat germ extract (Promega) was used to produce a 30 kDa protein that was reactive with an N-terminal antibody for MoMo30-plant and showed antiviral activity in a MAGI assay. Similarly, transfecting HEK293 cells with the expression plasmid produced a 30 kDa protein in the cell-free conditioned medium that was reactive with the N-terminal MoMo30-plant antibody, and the medium contained antiviral activity in a MAGI assay (see Fig. 7).

That MoMo30-plant could be a Hevamine A-like protein is consistent with its observed characteristics. Hevamine is one of several family members of plant chitinases and lysozymes produced as plant defensins against fungal infections [32]. Plants produce intracellular and extracellular chitinases of approximately 25–35 kDa [42]. We detected chitinase activity in MoMo30-plant (Additional file 1: Fig. S1). Hevamine is a 29 kDa protein that, according to the classification of Henrissat [43], is a class III chitinase from family 18. Members of this family have a conserved region of amino acids from residues 120 to 130 DGXDX**D**WEXP. The motif DDDE is highly conserved, as demonstrated by a psi BLAST search of over a thousand proteins with glycosyl transferase activity. One of the conserved aspartates (shown in bold) is replaced by arginine in Hevamine A-like proteins from both *M. balsamina* and *M. charantia*. This specific change was previously observed in the chitinase from *Aridopsis thaliania,* which maintains chitinase activity [44]. There are precedents for chitinases that can bind to glycoproteins. The lysine motif (LysM) is present in some chitinases from various sources [45]. A LysM motif is also present in the carbohydrate-binding protein CyanoVirin-N [46], which has been previously shown to have the ability to bind to gp120 on HIV and inhibit viral replication [47].

A hypothesis for the action of carbohydrate-binding agents (CBAs) has been proposed [23, 48]. Exposure of viruses to CBAs selects for variants expressing reduced numbers of carbohydrates on their surface (*i.e*., mutants with fewer binding opportunities for MoMo30-plant-plant). Selection of viruses with reduced glycosylated gp120 proteins allows for altered immunological responses and particles with impaired infectivity, which could result in longer-term suppression. MoMo30-plant appears to act as a CBA, and exposure to the protein may induce a change in glycan patterns on the surface of virions. Changes in gp120 induced by selection in the presence of MoMo30-plant may alter the antigenicity of gp120 and allow a robust neutralizing immune response to be mounted, as proposed in the CBA theory (26). Moreover, it is generally believed that the lack of a robust immune response to HIV-1 is that gp120 is heavily glycosylated (50). Furthermore, our observation that the monosaccharide mannose can block the antiviral effect of MoMo30-plant suggests that MoMo30-plant can bind to high-mannose glycans and select for viruses with reduced glycosylation on the surface [23].

Several other properties of MoMo30-plant make it a good candidate as a therapeutic for HIV-1. First, it has an IC_50_ of 2.8 nm, similar to the IC_50_ of approximately 20–40 nM for the commercially available fusion inhibitor Enfuvirtide [35]. Secondly, it acts directly on the gp120; inhibiting attachment negates the need for penetration into the cell. In addition, once MoMo30-plant is bound to gp120, it stays bound for extended periods (Figs. 8A, 2B). The biphasic nature of the Biacore sensorgram likely reflects our observation that MoMo30-plant tends to form multimers (Additional file 1: Fig. S2). The initial off rate could reflect the disruption of multimers.

In contrast, the longer off rate is the dissociation of MoMo30-plant in complex with gp120. This prolonged “off” rate could mean less protein needs to be delivered to the blood over time to reach and maintain therapeutic levels. In addition, since the virus is prevented from entering the cell, it minimizes the possibility of integration into the host genome, avoiding potential latent infection.

It has been suggested that fewer glycosylated amino acids on g120 might allow for an altered immune response, enhancing the opportunities for broad-based neutralizing antibodies to be produced. If so, our model for MoMo30-plant action predicts that individuals treated with plants containing MoMo30-plant should have high levels of broadly neutralizing antibodies. Such studies are underway and should provide a more complete understanding of the mechanism underlying this traditional African therapeutic approach.

## Conclusions

The medicinal plant *Momordica balsamina* contains a 30 kDa protein that we call MoMo30-plant-plant that has activity against strains of HIV-1. It acts as a fusion inhibitor by blocking the binding of gp120 to susceptible cells. MoMo30-plant binds to gp120 through its glycan residues. These data suggest MoMo30-plant-plant could represent a potential new inhibitor.

## Methods

### Plant extracts

The preparation of water extracts from plants has been previously described [33]. One hundred grams of dried and milled leaves from *Momordica balsamina* were extracted in 1L of distilled water overnight at 4 °C. The liquid extract was separated from the solid material through centrifugation at 4000×*g* for 30 min. at 4 °C. The resulting supernatant was filtered through Whatman filter paper (Cat# 3030) to remove particulates. The extract was then filter-sterilized by passing it through a 0.45-micron filter (Celltreat Cat # 229703) and was kept frozen at − 80 °C prior to lyophilization overnight.

### Isolation of MoMo30-plant

The lyophilized powder was dissolved in nuclease-free water (Invitrogen Cat# AM9938) to create a 15 mg/mL solution. The solution was passed through a 30kD molecular weight cutoff filter by centrifugation at 4000×*g* for 10 min (Amicon ultra-15 cat# UFC903024) to remove low molecular weight contaminants. Once a retentate of 1 to 1.5 mL was obtained, the solution was passed through a 0.22-micron syringe filter (Celltreate Cat# 229747) and stored at 4 °C before use (− 20 °C for long term storage). The retentate contained one protein MoMo30-plant-plant that was > 95% pure as determined by SDS-PAGE and stained with Coomassie Brilliant Blue R-250 (Bio-Rad Cat# 161-0400).

### Multinuclear activation of an indicator (MAGI) assay for infectivity

MAGI cell assays for infectivity were done as previously described [49, 50]. MAGI cells (U-373-MAGI-CXCR4_CEM_ glioblastoma cells, AIDS reagent program cat# ARP-3596) were grown to 90% confluence. Cells were infected with HIV-1_NL4-3_ (equivalent to 1 ng of p24; AIDS reagent program cat # 114). They were then fixed by the addition of 1% Formaldehyde (F-79–500 Fisher Chemicals) and 0.2% Glutaraldehyde (F-02957-1 Fisher Scientific) in PBS and stained in a solution that contained (14.25 mL PBS, 300 µL 0.2 M potassium ferrocyanide, 300 µL 0.2 M potassium ferricyanide, 15 µL 2 M MgCl_2_ and 150 µL X-gal stock (40 mg/mL in DMSO). Two mL solution was added to each well and incubated at 37 °C for 50 min. Cells were washed twice with PBS and counted using light microscopy. Infected cells were identified as those exhibiting the development of a blue color.

### Primary HIV-1 stocks

All HIV-1 strains (clade A to D) were obtained from the National Institutes of Health (NIH) AIDS Research and Reference Reagent Program (ARRRP) and were propagated at New Iberia Research Center by infecting human PBMCs 3 days after stimulation with 1.0 μg/mL ConA and culturing infected cells for 21 days after stimulation, replenishing media supplemented with 50 U/mL IL-2 twice a week. Viral supernatants were tested for HIV p24 antigen by ELISA kit (ABL Inc.) and supernatants with a high concentration were pooled. Virus-containing supernatants were clarified by centrifugation, sterile filtered and stored separately in 1-mL aliquots in liquid nitrogen.

### Neutralization assay in TZM-bl cells

The neutralization activity of MoMo30-plant-plant against each HIV-1 strain (clade A to D) was measured using a standard protocol of luciferase-based HIV-1 neutralization assay in TZM-bl cells (Montefiori, Duke University). Briefly, 50 µL of fivefold serial diluted MoMo30-plant-plant and 50 µL of 1 ng virus were preincubated for 1 h at 37˚C in a 96-well flat-bottom plate. Next, 100 µL of TZM-bl cells (1 × 10^4^/well) in 10% DMEM growth medium containing 15 µg/mL DEAE dextran (Sigma-Aldrich) were added to the preincubated each well, and the 96-well plates were incubated for 48 h. Assay controls included TZM-bl cells alone (cell control, no virus) and TZM-bl cells with virus only (virus control, no test reagent). At 48 h, the cells were lysed, and luciferase activity was measured using (Promega, Cat# E1501, 10 × 100 assays). on a BioTek Synergy HT multimode microplate reader The average background luminescence (RLU) from cell control wells was subtracted from the luminescence for each experimental well. The neutralization curves and 50% inhibitory concentration (IC_50_) were calculated and generated using GraphPad Prism (v7.01) software.

### Determination of the effect of MoMo30-plant and Enfuvirtide on HIV-1_NL4-3_ infectivity

We performed a dose–response curve on MoMo30-plant and Enfuvirtide (Sigma SML0934). We used concentrations of MoMo30-plant from 0.314 to 78.25 nM. Stock solution of MoMo30-plant protein (782.5 nM) was diluted to final concentrations of 0.314 nM, 0.609 nM, 1.22 nM, 2.44 nM, 4.88 nM, 9.77 nM,19.55 nM, 39.06 nM,and 78.25 nM. For Enfurvirtide we diluted at stock solution of 782.5 nM to final concentrations of 2.2 nM, 4.43 nM, 8.86 nM, 17.73 nM, 35.44 nM, 70.91 nM, 142.05 nM, 272.73 nM, and 568.00 nM. To each 1 mL of diluted inhibitor we added 5 µL of HIV-1_NL43_ (equivalent to 1 ng p24) and 10 µL of DEAE. The mixture was than added to 2 × 10^4^ MAGI cells and then incubated for 48 h at 37 °C. and blue cells were counted. The IC_50_ of MoMo30-plant was determined by curve fitting using the Hill equation and determined using the Dr. Fit program [34].

### Detection of MoMo30-plant in serum

To determine if the ingestion of plant extracts resulted in detectable levels of MoMo30-plant in the blood, two Rhesus macaques were given plant extracts using a scaled dosage to that typically given to humans. Basically, macaques were given the plant extracts with food. Two grams of plant was given twice a day for a period of six months. Blood samples were taken at 0 days up to 183 days. Plasma was tested by SDS -PAGE and Western blot.

### MTT assay of MoMo30-plant

To determine if MoMo30-plant had significant cellular toxicity at therapeutic levels, we exposed HEK 293 cells to concentrations of MoMo30-plant from 1 to 1000 nM and performed a mitochondrial toxicity test (MTT; Sigma Cat# CGD-1) according to the manufacturer's recommendations. Percent viability was determined by comparison to an untreated control.

### Stability studies on MoMo30-plant and its complex to gp120

To determine the heat stability of MoMo30-plant, we first subjected stock solutions of MoMo30-plant (4 ng/mL and 40 ng/mL) to temperatures from 25 to 120 °C for 30 min. After heating, the solution was mixed with 1 ng of HIV-1_NL4-3_ and was added to a MAGI cell assay, and blue cells were counted. In a separate study to determine the stability of complexes formed between virus and MoMo30-plant, we mixed virus equivalent to 1 ng p24 of HIV-1_NL4-3_ with a stock solution of MoMo30-plant (400 ng/mL) and kept the sample at 4 °C, we removed aliquots at time intervals of 5 min to 3 days, and centrifuged at 125,000 g through 20% sucrose cushion to remove free MoMo30-plant and tested its effect on infectivity by MAGI cell assay.

### N-terminal sequencing of MoMo30-plant

Edman degradation was performed on the plant-derived MoMo30-plant protein in two separate labs (Biosynthesis, Lewisville, TX, and Creative Proteomics, New York, NY). The analysis was performed on an ABI Procise 494HT (Thermo Fisher). The procedure determines the N-terminal amino acid sequence of proteins and peptides by the Edman degradation chemistry.

### RNAseq to determine the MoMo30-plant gene sequence

To help determine the gene sequence of MoMo30-plant we used RNAseq (Azenta Total RNA (~ 4 µg) purified from *M. balsamina* cells by the Trizol method was used for RNAseq on the Illumnina platform and the de novo.de novo transcriptome was assembled using Trinity software. The mRNA corresponding to the MoMo30-plant protein was determined by searching for the N-terminal sequence as determined by Edman degradation. Once a candidate DNA sequence had been determined we had the gene synthesized (Genscript) and cloned into the pGen-lenti vector which contains both a T7 promoter and a CMV promoter for expression in mammalian cells.

### Software for gene assembly and translation

BLAST searches were done at the national center for biotechnology information (NCBI) website. Comparisons of homology to various proteins and DNA sequences were made in SnapGene 6.0.2. Prediction of secondary structure was made at the Phyre2 structure prediction web portal [51].

### Coupled transcription/translation of MoMo30-plant gene

The cloned version of the synthesized gene was expressed in the wheat germ coupled transcription/translation system (TNT T7 coupled Wheat Germ Extract System Promega Cat# L4140) according to the manufacturer's recommendations with the following modification. Instead of adding [^35^S] methionine, we added 1 mM of unlabeled methionine to the mixture and detected protein using Western blot. Ten µL of the product was resolved on a 4–20% SDS-PAGE gel, and a Western blot was done using an antibody to the N-terminal peptide of MoMo30-plant. Ten µL of product was also tested in a MAGI cell infectivity assay to determine any antiviral effect. Since the pGen-lenti vector also contained a CMV promoter, we transfected HEK293 cells with the plasmid using Lipofectamine 3000 (ThermoFisher Scientific Cat # L3000008) following manufacturer's protocol and grew cells for 48 h. Conditioned medium was harvested, and cells were collected and lysed by using (Pierce RIPA buffer Cat# 89901). Ten µL of cell pellet and 20 µL of conditioned medium were resolved on a 4–20% SDS-PAGE gel and blotted with N-terminal antibody to MoMo30-plant. 100 µL of conditioned medium was tested by MAGI cell assay for antiviral effects.

### Fluorescent gp120 binding assay

Recombinant HIV-1 IIIB gp120 conjugated to FITC (ImmunoDX cat# 1001-F) was added to a suspension of 1 × 10^6^/mL Jurkat cells (AIDS reagent program ARP-177 E6-1 clone) allowed to interact for 2 h at 37 °C. Free gp120 was removed by centrifugation, and the cells were resuspended in PBS. A portion was stained with Hoechst stain, and the cells were viewed under a fluorescent microscope using a neutral density filter, a blue filter, and a FITC filter.

### Surface plasmon resonance (Biacore)

Surface plasmon resonance was done at the Biacore Molecular Interaction Shared Resource at Georgetown University. A Biacore T200 was used with a CM5 chip at 25 °C. Purified gp120-IIIB (Immuno Dx, 1 mg/mL) in 1 mM sodium acetate buffer at pH 5.5 was used as a ligand to immobilize onto FC2, FC3, and FC4 to the levels of 8850 RU, 283RU, and 2980RU, respectively. Standard amine coupling chemistry was used. HBS-P (10 mM Hepes, pH 7.4, 150 mM NaCl, 0.05% v/v surfactant P20) was used as the immobilization running buffer. Overnight kinetics were performed for MoMo30-plant binding to the ligand. Injected compound concentrations were 1–100 nM. Three 15 s pulses of 1:250 H_3_PO_4_ (v/v, ddH_2_O: H_3_PO_4_) were injected to regenerate the chip surface. All analyses were done in triplicate. The sensorgrams were obtained from overnight kinetics using 1:1 model fitting. In some experiments, gp120 was pre-treated with PNGase F (removes N-glycans) for 30 min at 50 °C before linkage to the chip surface. A control reaction was done with buffer alone. Three independent assays were done, each in triplicate.

### MoMo30-plant inhibition in the presence of mannose

To determine the effect of the monosaccharide mannose on the activity of MoMo30-plant, we did infectivity assays with 2 nM of MoMo30-plant in the presence of mannose in concentrations from 0.002 to 2 nM final concentration. and determined relative inhibition using a MAGI cell assay as described. In brief, 2 nM MoMo30-plant and different concentrations of D-Mannose (Sigma cat# M6020) were mixed. After that added, virus equivalent to 1 ng of p24 of HIV-1_NL4-3_ was incubated at room temperature for five minutes, added to MAGI cells, and determined relative inhibition was using a MAGI cell assay as described.

### Immunoblot

A rabbit antibody was produced (Genscript) from a 15-amino acid peptide with the N-terminal sequence of MoMo30-plant (GPIVTYGQNVNGELC). A separate rabbit antibody was made using a portion of the predicted sequence of the MoMo30-plant gene (LGGRSTSLRPGDC). The antibodies (at a dilution of 1:2000 for N-terminal ab; 1:3000 for the predicted sequence ab) were used to perform an immunoblot on purified protein resolved on a 4–20% SDS PAGE gel. The gels were transferred to 0.2 µm Nitrocellulose membrane using Bio-Rad Trans-Blot Turbo for 20 min and blocked with 0.5% skim milk made in Tris buffer saline with 0.1% tween 20 (TBST) for 1 h. The membrane was then incubated with primary antibody 1:2000 or 1:3000 in TBST overnight at 4 °C. The membranes were subjected to ten-minute washes with TBST and a wash with distilled water between each wash. Afterward, a secondary antibody (GE Healthcare goat anti-rabbit Cat# NA934V) was added at a dilution of 1:25,000 containing precision protein StrepTectin-HRP ( Bio-Rad Cat# 1610380) 1:10,000 and allowed to incubate for 1 h at room temperature. After this, the membrane was washed three times as previously, and chemiluminescent substrate (SuperSignal West Femto Thermo Scientific Cat# 34096) was added and incubated for 5 min. The blot was visualized by a chemiluminescent imager (ThermoFisher iBright 1500).

## Supplementary Information

**Additional file 1: Fig. S1.** MoMo30-plant has chitinase activity. To determine if MoMo30-plant had chitinase activity, 5 µL of a 200 nM solution of MoMo30-plant was added to three different substrates (Sigma; cat # CS1030) in 100 µL of total volume a positive control is included for each substrate: 4-Methylumbelliferyl N,N′-diacetyl-β-D-triacetylchitotriose (Endo; endochitinase activity), 4-Methylumbelliferyl N,N′-diacetyl-β-D-chitobioside (Chito; exochitinase activity), and 4-Methylumbelliferyl N,N′-diacetyl-β-D-glucosaminide (Gluc; exochitinase activity). The assay is performed in an acidic environment (pH ~ 5.0) at 37 °C for 30 min. The assays were done in triplicate. The activity was measured as fluorescence and converted to activity in mg/mL. Fluorecent substrates require the least amount of time and were the most sensitive of the common methods used for chitinase activity [52].

**Additional file 2: Fig. S2.** MoMo30-plant forms multimers on a native PAGE gel (4–20% TGX gels from Bio-Rad). The gel was then stained with Coomassie brilliant blue.

## Data Availability

No data sets were generated as a part of this study.
