# Supplementary figures and images for: Anti-human immunodeficiency virus-1 activity of MoMo30 protein isolated from the traditional African medicinal plant Momordica balsamina
Source: Virol J. 2023 Mar 22;20:50. doi: 10.1186/s12985-023-02010-5 (PMC10035133; doi:10.1186/s12985-023-02010-5)

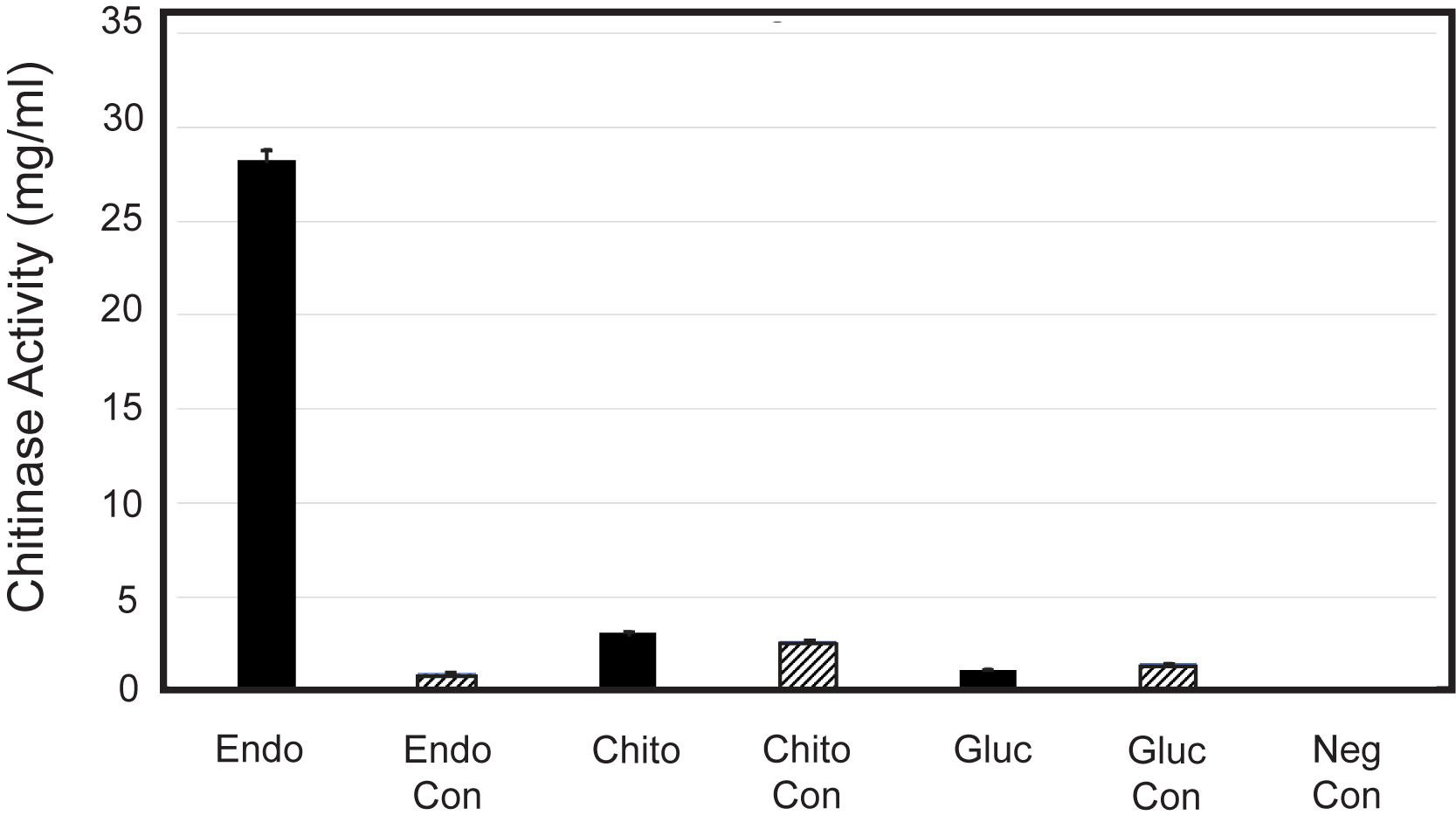

Supplement: Supplementary file 1 — Additional file 1: Fig. S1. MoMo30-plant has chitinase activity. To determine if MoMo30-plant had chitinase activity, 5 µL of a 200 nM solution of MoMo30-plant was added to three different substrates (Sigma; cat # CS1030) in 100 µL of total volume a positive control is included for each substrate: 4-Methylumbelliferyl N,N′-diacetyl-β-D-triacetylchitotriose (Endo; endochitinase activity), 4-Methylumbelliferyl N,N′-diacetyl-β-D-chitobioside (Chito; exochitinase activity), and 4-Methylumbelliferyl N,N′-diacetyl-β-D-glucosaminide (Gluc; exochitinase activity). The assay is performed in an acidic environment (pH ~ 5.0) at 37 °C for 30 min. The assays were done in triplicate. The activity was measured as fluorescence and converted to activity in mg/mL. Fluorecent substrates require the least amount of time and were the most sensitive of the common methods used for chitinase activity [52]. [file 12985_2023_2010_MOESM1_ESM.tif]

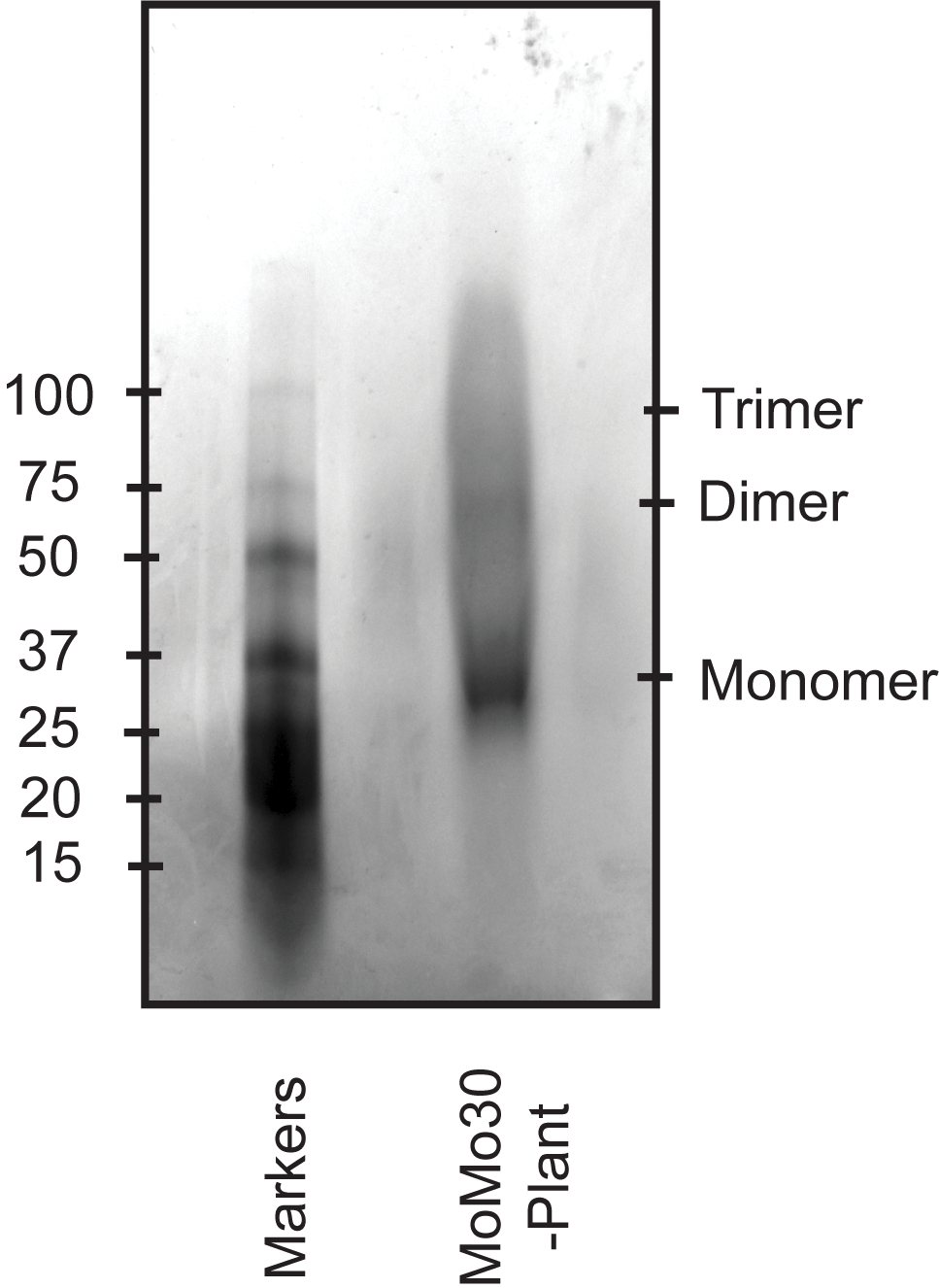

Supplement: Supplementary file 2 — Additional file 2: Fig. S2. MoMo30-plant forms multimers on a native PAGE gel (4–20% TGX gels from Bio-Rad). The gel was then stained with Coomassie brilliant blue. [file 12985_2023_2010_MOESM2_ESM.tif]
